# Is It Possible to Predict False-Positive Exercise Stress Echocardiography Results by Measuring the Left Atrial Antero-Posterior Diameter?

**DOI:** 10.7759/cureus.53857

**Published:** 2024-02-08

**Authors:** Andrea Sonaglioni, Gian Luigi Nicolosi, Michele Lombardo

**Affiliations:** 1 Cardiology, MultiMedica Istituto di Ricovero e Cura a Carattere Scientifico (IRCCS), Milan, ITA; 2 Cardiology, Policlinico San Giorgio, Pordenone, ITA

**Keywords:** left atrial anteroposterior diameter, exercise stress echocardiography, false positive, chest wall conformation, coronary artery disease

## Abstract

Background: Left atrial (LA) size is a well-known prognostic determinant in the setting of coronary artery disease (CAD). No previous study has evaluated LA antero-posterior (A-P) diameter as a potential screening method for identifying individuals with a low probability of CAD. We aimed to assess the influence of LA A-P diameter adjusted for chest wall conformation (A-P thoracic diameter) on the occurrence of false-positive (FP) results on exercise stress echocardiography (ESE) in patients with suspected CAD.

Methods: All consecutive patients who had undergone coronary angiography at MultiMedica IRCCS (via San vittore 12, 20123, Milan, Italy) within two months from a positive ESE over a seven-year period were retrospectively analyzed. All patients underwent LA A-P diameter/A-P thoracic diameter ratio assessment, resting transthoracic echocardiography, and subsequent ESE. The primary endpoint was FP-ESE, defined as a positive ESE with no evidence of obstructive CAD (≥70% stenosis in any epicardial coronary artery) on subsequent coronary angiography.

Results: A total of 160 patients (64.4±13.0 years, 56.9% females) with a positive ESE were retrospectively analyzed. In light of coronary angiography results, 129 patients (80.6%) had an obstructive CAD, while 31 (19.4%) did not (FP). On the multivariate logistic regression analysis, the LA A-P diameter/A-P thoracic diameter ratio (odds ratio (OR) 0.42, 95% confidence interval (CI) 0.31-0.57) showed a strong inverse correlation with the primary endpoint. An LA A-P diameter/A-P thoracic diameter ratio ≤0.25 had 100% sensitivity and 85% specificity for predicting FP-ESE results (area under the curve (AUC) = 0.94). A strong linear correlation was demonstrated between the LA A-P diameter and A-P thoracic diameter (r = 0.85), whereas the correlation between the LA volume index and A-P thoracic diameter was moderate (r = 0.47).

Conclusions: Echocardiographic assessment of the LA A-P diameter adjusted for the A-P thoracic diameter may allow clinicians to identify, among individuals with suspected CAD, those at lower risk of obstructive CAD.

## Introduction

Traditionally, the left atrial (LA) antero-posterior (A-P) diameter, obtained from the parasternal long-axis view using M-mode or two-dimensional (2D) transthoracic echocardiography (TTE), has been the most widely used dimensional measurement of left atrium, due to its excellent reproducibility [1-5]. However, assessment of the LA size using only the A-P diameter may not always describe an accurate picture of the LA size [6,7]. Indeed, when the left atrium enlarges, not all its dimensions change similarly and an asymmetric LA enlargement may often occur. Notably, the LA enlargement in the A-P direction is constricted by the presence of the spine and sternum, and, consequently, most LA enlargement tends to occur in the superior-inferior direction [8] Therefore, the 2D-derived LA volume may allow to obtain a more accurate measure of the true size of left atrium [9,10]. In addition, compared with the LA A-P diameter, LA volumetric measurements are a stronger predictor of outcome in patients with coronary artery disease (CAD) [11-17] and other cardiac diseases [18-25]. Therefore, measurement of LA volumes through the biplane disk summation technique is strongly recommended by the American Society of Echocardiography 2015 chamber quantification guidelines [26]. On the other hand, diagnostic and prognostic data of three-dimensional echocardiography LA volumes are limited [27-29].

Despite the prognostic relevance of LA size in the CAD setting, its evaluation in terms of prognostic risk stratification of patients with suspected CAD is not adequately considered in daily practice. It is noteworthy that a large proportion of patients with suspected CAD are referred to echo labs to perform electrocardiographic (ECG) and/or echocardiographic stress testing for “rarely appropriate” reasons [30-32] according to the appropriate use criteria [33] and that, during the last few years, there is not a significant temporal trend toward rising rates of appropriateness for noninvasive provocative testing [34,35].

During the last few years, our study group developed the modified Haller index (MHI, the ratio of chest transverse diameter over the distance between the sternum and spine) [36], a nonradiological modality for assessing chest wall conformation. This technique allows to noninvasively quantify the degree of anterior chest wall deformity, with increasing MHI value from the flat chest and/or mild concave-shaped chest wall to severe forms of pectus excavatum (PE). A strong inverse correlation between the MHI magnitude and obstructive CAD and/or adverse cardiovascular events was demonstrated in individuals with various types of anterior chest wall deformity and in healthy individuals with mitral valve prolapse (MVP) [37-39].

We previously demonstrated that chest wall abnormalities may influence exercise stress echocardiography (ESE) results [40,41]. Even if the LA A-P diameter is obviously correlated with the distance between the sternum and spine, as far as we know, to date, no researcher has previously quantified the degree of correlation between the LA A-P diameter and the anterior chest wall conformation. To obtain an objective measure of the relationship between the LA A-P diameter and the anterior chest wall conformation, our study group conceived the ratio of the LA A-P diameter to the A-P thoracic diameter.

In light of our previous findings [40,41], we hypothesized that individuals with a lower LA A-P diameter/A-P thoracic diameter ratio might have a significantly lower probability of obstructive CAD and a significantly increased probability of false-positive (FP)-ESE results, in comparison to those with a greater A-P thoracic diameter/LA A-P diameter ratio, due to the good cardiovascular prognosis associated with a small LA cavity size, as commonly observed in individuals with concave-shaped chest wall and/or PE.

Accordingly, the present study was primarily designed to evaluate the influence of the LA A-P diameter/A-P thoracic diameter ratio on the occurrence of FP-ESE results in patients with suspected CAD.

## Materials and methods

Study population

All consecutive patients who had undergone coronary angiography at MultiMedica IRCCS (via San vittore 12, 20123, Milan, Italy) within two months from a positive ESE between June 2014 and May 2020 were retrospectively analyzed.

ESE was performed for one of the following indications: 1) chest pain of suspected cardiac origin, 2) resting/stress test ECG abnormalities, and/or 3) dyspnea of unclear origin.

Exclusion criteria were as follows: 1) patients who did not undergo a semisupine ESE for suspected CAD, 2) patients who resulted negative for myocardial ischemia on ESE, and 3) patients who did not undergo LA A-P diameter/A-P thoracic diameter ratio assessment.

The following parameters were obtained from the patients’ medical records: age, gender, body surface area (BSA), resting systolic blood pressure (SBP) and diastolic blood pressure, resting peripheral arterial oxygen saturation (SaO_2_), presence of relevant cardiovascular risk factors (smoking, hypertension, type-2 diabetes, and dyslipidemia), estimated glomerular filtration rate [42], pre-test probability (PTP) of CAD calculated according to the 2019 ESC guidelines for the diagnosis and management of chronic coronary syndrome [43], and pharmacological treatment (if any). ECG data included cardiac rhythm, heart rate, left ventricular (LV) depolarization, and repolarization pattern.

During the same day, each patient underwent physical examinations, resting 2D-TTE, and subsequent ESE.

All procedures were in accordance with the ethical standards of our Institutional Research Committee and with the 1964 Helsinki Declaration and its later amendments or comparable ethical standards. The present study was based on a retrospective analysis of examinations already performed, justified by good clinical practice. A written informed consent was obtained from all participants (Committee’s reference number: 465.2021).

Conventional transthoracic echocardiography

All echocardiographic examinations were performed by the same cardiologist (A.S.) with specific training and experience in cardiovascular echocardiography, using a commercially available Philips Sparq ultrasound machine (Philips, Andover, Massachusetts, USA) with a 2.5 MHz transducer, according to the recommendations of the American Society of Echocardiography and the European Association of Cardiovascular Imaging [26,44]. Following resting, echo Doppler measurements were obtained: LV end-diastolic dimensions; relative wall thickness (RWT); left ventricular mass index (LVMi) calculated by the Devereux’s formula; left ventricular ejection fraction (LVEF) measured by the biplane modified Simpson's method [26]; LA A-P diameter and left atrial volume index (LAVi) as linear and volumetric indices of the LA size, respectively; LV diastolic function assessed by the E/A ratio and E/average e’ ratio [44]; degree of concomitant valvular heart disease assessed according to the AHA/ACC recommendations [45]; right ventricular end-diastolic diameter; right ventricular longitudinal function measured by tricuspid annular plane systolic excursion (TAPSE); and finally systolic pulmonary artery pressure (SPAP) calculated by Bernoulli's equation, where SPAP = 4x tricuspid regurgitation velocity (TRV)2 + right atrial pressure; the latter was estimated by inferior vena cava diameter and its inspiratory collapse [46].

A-P thoracic diameter assessment

**Figure 1:**
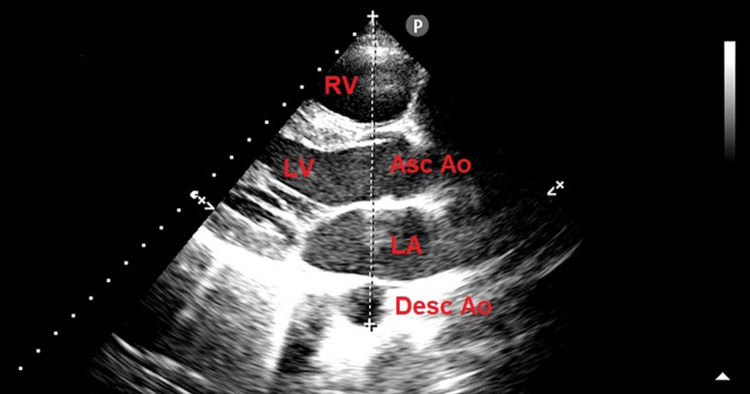
Echocardiographic assessment of the anteroposterior thoracic diameter. Transthoracic echocardiography, parasternal long-axis view: The white dotted line indicates the anteroposterior thoracic diameter, measured as the distance between the true apex of the sector and the anterior surface of the vertebral body. The latter is identified by using, as a reference point, the posterior wall of the descending thoracic aorta, visualized behind the left atrium. Ao, aorta; Asc, ascending; Desc, descending; LA, left atrium; LV, left ventricle; RV, right ventricle

The A-P thoracic diameter is measured from the parasternal long-axis view, as the distance between the true apex of the sector and the anterior surface of the vertebral body. The latter is identified by using, as a reference point, the posterior wall of the descending thoracic aorta, visualized behind the left atrium [36] (Figure 1).

Exercise stress echocardiography

The patients underwent incremental exercise test on a semi-supine bicycle ergometer (Ergoselect 1200 ELP; Ergoline, Bitz, Germany), with initial work-load set at 25 W, increased by 25 W every two minutes. A 12-lead ECG was continuously monitored. Heart rate, blood pressure (BP), peripheral arterial oxygen saturation, and echo Doppler measurements (comprehensive of the LVEF, E/average e’ ratio, degree of concomitant mitral regurgitation, TAPSE, and finally SPAP assessment) were collected at rest, every two minutes during the test, at peak exercise, and during recovery. The ischemic ST-segment response was defined as occurrence of ≥0.1 mV horizontal or downsloping ST-segment depression below the resting level (measured 60 ms after the J-point), ≥0.2 mV upsloping depression (measured 80 ms after J-point), or ≥0.1 mV elevation [47]. Positive ESE was defined as new or worsened wall motion abnormalities during stress, as indicated by an increase in wall motion score ≥1 grade in ≥2 segments [48].

Statistical analysis

The primary endpoint of the study was to identify the parameters independently associated with an FP-ESE result. The latter was defined as a positive ESE with no evidence of obstructive CAD on subsequent coronary angiography. Conversely, a true-positive (TP) ESE result was defined as a positive ESE with obstructive CAD according to subsequent coronary angiography. Obstructive CAD was defined as 70% or more diameter narrowing in the lumen of the left anterior descending (LAD), left circumﬂex, and right coronary artery or 50% or more in the lumen of the left main coronary artery.

Each continuous variable was checked through the Shapiro-Wilk test, and all data were determined to be normally distributed. For the whole study population, continuous data are summarized as mean ± standard deviation, while categorical data are presented as percentages.

The correlation between between the LA A-P diameter and the A-P thoracic diameter and between the LAVi and the A-P thoracic diameter, in the whole study group, was evaluated by using Pearson’s correlation coefﬁcient.

Univariate logistic regression analysis was performed to evaluate the effect of the main variables that were hypothesized to be related with the occurrence of an FP-ESE result in the whole study group. According to the “one-in-10 rule” (one predictive variable for every 10 events), only the following variables were included in the logistic regression analysis: female sex (as a demographic variable), PTP (as a clinical variable), LA A-P diameter/A-P thoracic diameter ratio (as a resting anthropometric/echocardiographic variable), and finally peak exercise SBP (as an ESE-related variable). For each variable investigated, correspondent ORs with 95% CIs were calculated. Those variables with a statistically significant association on the univariate analysis were thereafter included in the multivariate logistic regression model. The receiver operating characteristic (ROC) curve analysis was performed to establish the sensitivity and speciﬁcity of the main statistically significant continuous variable for predicting the FP-ESE results in our study group. The area under the curve (AUC) was estimated.

To evaluate intra- and inter-observer variability in the assessment of the LA A-P diameter and A-P thoracic diameter, both variables were re-assessed in a subgroup of 15 randomly selected individuals included in the present study by the same cardiologist who performed all echocardiographic examinations (A.S.) and by a second one (M.L.). The analyses were performed in a blinded manner. Both raters chose the frame on which to perform each measurement. We used the intraclass correlation coefficient (ICC) with its 95% CI, as a statistical method for assessing intra- and inter-observer measurement variabilities. An ICC of 0.70 or more was considered to indicate acceptable reliability.

Statistical analysis was performed with IBM SPSS Statistics for Windows, version 28 (released 2021; IBM Corp., Armonk, New York, United States), with two-tailed P values below 0.05 deemed statistically significant.

## Results

A total of 160 patients (mean age 64.4 ± 13.0 years, 56.9% females) were retrospectively analyzed. All demographic, anthropometric, clinical, resting hemodynamics, electrocardiographic, and echo Doppler parameters detected in the whole study population are summarized in Table 1.

**Table 1:** Demographic, anthropometric, electrocardiographic, echo Doppler, and clinical parameters detected in the whole study population, at basal evaluation. ACE-i, angiotensin-converting enzyme inhibitors; AF, atrial fibrillation; ARBs angiotensin II receptor blockers; A-P, antero-posterior; BSA, body surface area; DBP, diastolic blood pressure; ECG, electrocardiographic; eGFR, estimated glomerular filtration rate; ESE, exercise stress echocardiography; HR heart rate; LA, left atrial; LAVi, left atrial volume index; LBBB, left bundle branch block; LVEDVi, left ventricular end-diastolic volume index; LVEF, left ventricular ejection fraction; LVMi, left ventricular mass index; MVP, mitral valve prolapse; MR, mitral regurgitation; NS-STT, non-specific ST-segment and T-wave; PTP, pre-test probability; RVEDD, right ventricular end-diastolic diameter; RWT, relative wall thickness; SaO2, arterial oxygen saturation; SBP, systolic blood pressure; SPAP, systolic pulmonary artery pressure; TAPSE, tricuspid annular plane systolic excursion; *Calculated in patients with sinus rhythm; **Calculated in all patients.

	All patients (n = 160)
Demographics and anthropometrics	
Age (years)	64.4 ± 13.0
Female sex (n, %)	91 (56.9)
BSA (m^2^)	1.80 ± 0.19
A-P thoracic diameter (cm)	14.6 ± 1.8
Cardiovascular risk factors	
Hypertension (n, %)	103 (64.4)
Type 2 diabetes mellitus (n, %)	29 (18.1)
Smoking (n, %)	64 (40.0)
Dyslipidemia (n, %)	68 (42.5)
eGFR(ml/min/m^2^)	75.4 ± 23.6
Indications for ESE	
Chest pain of suspected cardiac origin (n, %)	39 (24.4)
Resting/stress test ECG abnormalities (n, %)	49 (30.6)
Dyspnea of unclear origin (n, %)	72 (45.0)
PTP (%)	24.3 ± 7.1
Resting hemodynamics	
SBP (mmHg)	129.5 ± 16.5
DBP (mmHg)	81.0 ± 8.3
SaO_2_ (%)	97.2 ± 1.5
Resting ECG parameters	
HR (bpm)	73.6 ± 12.7
AF (n, %)	6 (3.7)
NS-STT abnormalities (n, %)	48 (30.0)
LBBB (n, %)	17 (10.6)
Resting echo Doppler parameters	
LVEDVi (ml/m^2^)	46.2 ± 8.2
RWT	0.40 ± 0.05
LVMi (g/m^2^)	95.3 ± 22.5
LVEF (%)	59.1 ± 4.6
E/A ratio*	0.90 ± 0.22
Average E/e’ ratio**	9.0 ± 2.6
LA A-P diameter (mm)	37.9 ± 5.2
A-P thoracic diameter/LA A-P diameter	3.90 ± 0.45
LAVi (ml/m^2^)	35.2 ± 7.8
RVEDD (mm)	27.8 ± 5.3
TAPSE (mm)	22.3 ± 1.9
SPAP (mmHg)	27.9 ± 5.4
Mild-to-moderate MR due to MVP (n, %)	43 (26.9)
Medical treatment	
Antiplatelets (n, %)	82 (51.2)
ACE-i/ARBs (n, %)	76 (47.5)
Calcium channel blockers (n, %)	38 (23.7)
Beta blockers (n, %)	80 (50.0)
Diuretics (n, %)	23 (14.4)
Statins (n, %)	59 (36.9)

Our study group had a moderate-to-high prevalence of hypertension (64.4%), a moderate prevalence of smoking (40%) and dyslipidemia (42.5%), and a lower prevalence of type 2 diabetes (18.1%). The main indication for stress testing was dyspnea of unclear origin (45% of cases), followed by resting/stress test ECG abnormalities (30.6% of cases) and chest pain of suspected cardiac origin (24.4% of cases). The PTP of CAD, based on age, sex, and symptoms, was intermediate. Analysis of hemodynamics showed normal BP values and normal peripheral SaO_2_ in the whole study group. On resting ECG, only 3.7% of the patients had atrial fibrillation, whereas nonspecific ST-segment and T-wave abnormalities were detected in 30% of the patients. Overall, TTE showed a normal cardiac chamber cavity size, normal biventricular systolic function (as assessed by the LVEF and TAPSE, respectively), a first degree of diastolic dysfunction, with the E/average e’ ratio in the “gray zone” between 8 and 13, and finally a normal SPAP; a mild-to-moderate mitral regurgitation (MR) due to MVP was detected in 26.9% of the patients. Concerning medical treatment, antiplatelets, angiotensin-converting-enzyme (ACE) inhibitors, and beta blockers were prescribed in approximately half of the patients, whereas calcium channel blockers, diuretics, and statins were much less commonly used.

Main hemodynamics, ECG, echo Doppler changes, and symptoms recorded at peak exercise in the whole study population are reported in Table 2.

**Table 2:** Main hemodynamics, electrocardiographic, and echo Doppler changes and symptoms detected at peak exercise in the whole study population. DBP, diastolic blood pressure; DP, double product; HR, heart rate; LAD, left anterior descending; LCx, left circumflex; LVEF, left ventricular ejection fraction; MR, mitral regurgitation; RCA, right coronary artery; SaO2, oxygen saturation; SBP, systolic blood pressure; SPAP, systolic pulmonary artery pressure; SVPBs, supraventricular premature beats; TAPSE, tricuspid annular plane systolic excursion; VPBs, ventricular premature beats; WMSI, wall motion score index; *Observed in patients with sinus rhythm; **Measured in all patients.

	All patients (n = 160)
Hemodynamics at peak exercise	
Watts reached	95.9 ± 31.4
Female sex (n, %)	96.7 ± 1.9
SBP (mmHg)	170.1 ± 22.2
DBP (mmHg)	88.2 ± 12.3
HR (bpm)	120.9 ± 20.3
SBP (mmHg)	170.1 ± 22.2
Percentage of HR maximum reached (%)	77.2 ± 10.7
DP (mmHg × bpm)	20.714.6 ± 4847.8
ECG parameters at peak exercise	
Isolated SVPBs (n, %)	27 (16.9)
Isolated VPBs (n, %)	53 (33.1)
Upsloping ST depression ≥2 mm (n, %)	50 (31.2)
Downsloping ST depression ≥1 mm (n, %)	21 (13.1)
Horizontal ST depression ≥1 mm (n, %)	12 (7.6)
Echo-Doppler parameters at peak exercise	
LVEF (%)	47.8 ± 6.1
WMSI	1.71 ± 0.37
Dyssynergy in the LAD territory (n, %)	85 (53.1)
Dyssynergy in the RCA territory (n, %)	25 (15.6)
Dyssynergy in the LCx territory (n, %)	38 (23.8)
Multivessel distribution (n, %)	12 (7.5)
Pseudonormalization of the E/A ratio (n, %)*	53 (33.1)
Average E/e’ ratio**	13.4 ± 5.9
Severe MR (n, %)	15 (9.4)
TAPSE (mm)	27.7 ± 5.8
SPAP ≥60 mmHg (n, %)	34 (21.2)
EXERCISE INDUCED-SYMPTOMS	
Dyspnea (n, %)	37 (23.1)
Typical chest pain (n, %)	24 (15)
Atypical chest pain (n, %)	13 (8.1)
Palpitations (n, %)	21 (13.1)

BP values showed a physiological response to dynamic exercise. An exercise-induced upsloping ST depression ≥2 mm was observed more frequently (31.2% of cases) than downsloping (13.1% of cases) and horizontal (7.6% of cases) ST depression. Isolated ventricular premature beats (33.1%) were more frequently recorded than isolated supraventricular premature beats (16.9%).

An exercise-induced mild-to-moderate depression of the LVEF was diagnosed in the whole study population. Dyssynergy in the LAD artery territory was much more frequently detected (53.1% of cases), followed by dyssynergy in the left circumflex artery (LCx) territory (23.8% of cases), dyssynergy in the right coronary artery (RCA) territory (15.6% of cases) and global LV hypokinesis as for multivessel distribution (7.5% of cases). An exercise-induced pseudonormalization of the E/A ratio (second degree of diastolic dysfunction) was observed in 33.1% of patients. A severe exercise-induced MR was diagnosed in 9.4% of cases, whereas a severe exercise-induced pulmonary hypertension (SPAP ≥60 mmHg) was detected in 21.2% of cases. The most common symptom observed during ESE was dyspnea (23.1% of cases), followed by typical chest pain (15% of cases) and palpitations (13.1% of cases).

Coronary angiography results

Coronary angiography was performed at 19.7 ± 7.1 days from ESE. Among the 160 patients with positive ESE, 129 (80.6%) had an obstructive CAD (TP), while 31 (19.4%) not (FP). More than half of patients (53% of the total) were diagnosed with critical atherosclerotic stenosis of the LAD; severe RCA and LCx stenosis were detected in 26% and 15% of patients, respectively, while 6% of patients were found with a multi-vessel disease. 114 patients (88.0%) underwent percutaneous coronary intervention, while the remaining 15 (12.0%) were treated with coronary artery bypass grafting.

Predictors of FP-ESE results

On univariate logistic regression analysis, female sex (OR 3.98, 95%CI 1.53-10.3, P = 0.005) was linearly correlated with the primary endpoint, whereas the LA A-P diameter/A-P thoracic diameter ratio (OR 0.41, 95%CI 0.30-0.56, P <0.001) showed a strong inverse correlation with FP-ESE result. On multivariate analysis, LA A-P diameter/A-P thoracic diameter ratio (OR 0.42, 95%CI 0.31-0.57, P <0.001) retained statistical significance (Table 3).

**Table 3:** Univariate and multivariate logistic regression analyses performed to identify the main independent predictors of FP-ESE results in the whole study group. Significant P values are in bold. A-P, antero-posterior; ESE, exercise stress echocardiography; FP, false positive; LA, left atrial; PTP, pre-test probability; SBP, systolic blood pressure.

	Univariate logistic regression analysis			Multivariate logistic regression analysis		
	OR	95% CI	P value	OR	95% CI	P value
Female sex	3.98	1.53-10.3	0.005	1.38	0.34-5.59	0.65
PTP (%)	0.97	0.91-1.03	0.37			
A-P thoracic diameter/LA A-P diameter	0.41	0.30-0.56	<0.001	0.42	0.31-0.57	<0.001
Peak exercise SBP (mmHg)	1.00	0.99-1.02	0.56			

**Figure 2:**
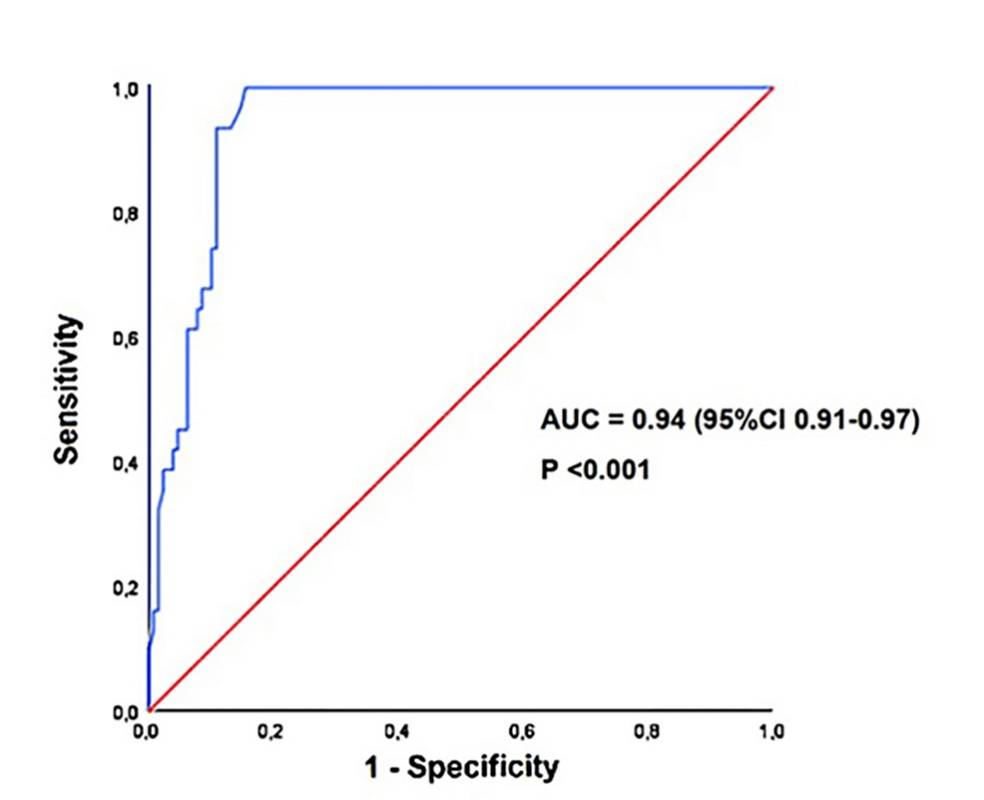
ROC curve analysis performed to determine the sensitivity and specificity of the LA A-P diameter/A-P thoracic diameter ratio for predicting FP-ESE results in our study group. A-P, antero-posterior; AUC, area under the curve; ESE, exercise stress echocardiography; FP, false positive; LA, left atrial; ROC, receiver operating characteristics.

ROC curve analysis showed that a LA A-P diameter/A-P thoracic diameter ratio ≤0.25 had 100% sensitivity and 85% specificity for predicting FP ESE results (AUC = 0.94, 95%CI 0.91-0.97) (Figure 2).

**Figure 3:**
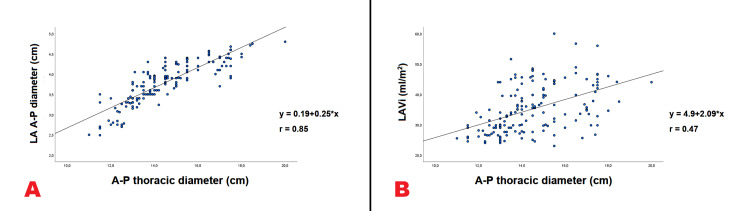
Correlation between the LA A-P diameter and the A-P thoracic diameter (Panel A) and between LAVi and the A-P thoracic diameter (Panel B), in the whole study group, evaluated by using Pearson’s correlation coefﬁcient. A-P, antero-posterior; LA, left atrial; LAVi, left atrial volume index.

A strong linear correlation was demonstrated between LA A-P diameter and the A-P thoracic diameter (r = 0.85) (Figure 3, Panel A), whereas the correlation between LAVi and the A-P thoracic diameter was moderate (r = 0.47) (Figure 3, Panel B).

**Figure 4:**
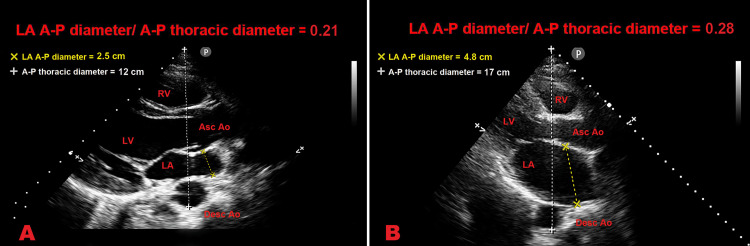
Examples of the LA A-P diameter/A-P thoracic diameter ratio obtained in individuals with concave-shaped chest wall (Panel A) and normal chest wall conformation (Panel B) respectively, included in the present study. In light of our findings, the magnitude of the LA A-P diameter/A-P thoracic diameter ratio obtained in the participant with a concave-shaped chest (≤0.25), as that measured in Panel A, identified an individual with a low probability of obstructive CAD and high probability of FP-ESE result. Ao, aorta; A-P, antero-posterior; Asc, ascending; CAD, coronary artery disease; Desc, descending; ESE, exercise stress echocardiography; FP, false positive; LA; left atrium; LV, left ventricle; RV, right ventricle.

Figure 4 illustrates examples of LA A-P diameter/A-P thoracic diameter ratio obtained in individuals with concave-shaped chest wall (Panel A) and normal chest wall conformation (Panel B) respectively, included in the present study.

Measurement variability

Intra- and inter-observer agreement in the assessment of LA A-P diameter was 0.95 (0.86-0.98) and 0.88 (0.69-0.96) respectively, whereas intra- and inter-observer agreement in the measurement of A-P thoracic diameter was 0.97 (0.91-0.99) and 0.95 (0.87-0.98) respectively (Table 4).

**Table 4:** Intra- and inter-observer variability analysis of LA A-P diameter and A-P thoracic diameter measurement performed in a subgroup of 15 randomly selected individuals, included in the present study. A-P, antero-posterior; CI, confidence interval; ICC, intraclass correlation coefficient; LA, left atrial.

	LA A-P diameter (cm)			A-P thoracic diameter (cm)		
PATIENT LIST	INITIAL MEASUREMENT	REMEASUREMENTS		INITIAL MEASUREMENT	REMEASUREMENTS	
		Rater 1	Rater 2		Rater 1	Rater 2
A.S.	3.05	2.80	2.60	14.5	14.1	14
A.L.	3.50	3.20	3.00	13.7	13.5	13
E.G.	3.25	2.90	2.70	13.1	13	12.8
G.G.	3.00	2.90	2.80	14.5	14	13.8
M.Z.	3.10	3.00	2.90	16.5	16.1	16
F.C.	3.40	3.30	3.20	14.5	14.1	14
G.C.	4.15	3.95	3.90	13.7	13.1	13
E.R.	3.35	3.30	3.25	14.5	14.2	14.6
S.M.	3.55	3.60	3.70	17	17.1	17.3
M.P.	3.90	4.00	4.10	15.5	15.3	15.6
C.F.	3.50	3.46	3.40	15.5	15.6	15.7
N.R.	4.00	3.95	3.85	13.7	13.9	13.5
D.B.	3.80	3.70	3.60	15	14.8	14.6
G.S.	3.60	3.50	3.40	13.7	13.9	13.4
S.R.	4.10	3.90	3.80	15	15.2	15.3
ICC (95%CI)		0.95 (0.86-0.98)	0.88 (0.69-0.96)		0.97 (0.91-0.99)	0.95 (0.87-0.98)

## Discussion

The present study that analyzed a retrospective cohort of individuals with positive ESE who underwent subsequent coronary angiography demonstrated that the LA A-P diameter/A-P thoracic diameter ratio was the main independent predictor of FP-ESE result. This parameter showed an incremental predictive value over the female sex, PTP, and peak exercise SBP. An LA A-P diameter/A-P thoracic diameter ratio ≤0.25 showed the highest sensitivity and specificity for detecting false positivity on ESE. In other terms, the lowest was the LA A-P diameter/A-P thoracic diameter ratio, and the greatest was the probability of FP-ESE result. A possible explanation for these findings may be related to the lower prevalence of obstructive CAD among individuals with a narrow LA A-P diameter secondary to a narrow A-P thoracic diameter, who are commonly women with a low prevalence of the main cardiovascular risk factors, atypical chest pain, and moderate-to-low PTP of CAD. These individuals are frequently diagnosed with nonspecific ST-segment and T-wave abnormalities on resting ECG, with exercise-induced upsloping ST depression ≥2 mm and with mild-to-moderate MR due to MVP. Given that a high incidence of concomitant MVP has been frequently reported in individuals with thoracic skeletal abnormalities, such as PE, "straight back syndrome," and scoliosis [49-51], previous authors have hypothesized that the constant mechanical stress and the compression imposed by a narrow A-P thoracic diameter may cause a distortion of mitral valve annulus with consequent MVP/MR, in the absence of any intrinsic myocardial dysfunction or obstructive CAD [52-54].

As expected, we found a strong linear correlation between the LA A-P linear dimension and the anterior chest wall conformation: the narrowest was the A-P thoracic diameter and the lowest was the LA A-P diameter size, thus suggesting a strong influence of the anterior chest wall conformation on LA A-P linear measurement. The anterior chest wall conformation showed a weaker correlation with LAVi, likely due to technical reasons. Indeed, LAVi measurement may be underestimated in the case of suboptimal visualization of LA endocardial borders from the four-chamber and two-chamber apical views or overestimated by the inadvertent incorporation of a part of the pulmonary veins or left atrial appendage in the LA volumetric measurement [55]. Moreover, potential measurement errors are commonly amplified during LA volume calculation, while LA linear measurements are more precise and reproducible.

As far as we know, this is the first study that correlated an LA linear measurement with the occurrence of FP-ESE result. According to literature data, the main predictors of FP-ESE results are female sex, low PTP of CAD, hypertensive response to exercise, resting regional wall motion abnormalities (particularly the left bundle branch block), and exercise-induced Takotsubo-like pattern [56]. Our findings revealed that another potential mechanism capable of inducing LV wall motion abnormalities, hence possibly yielding FP-ESE results, is represented by abnormalities of chest anatomy, particularly anterior chest wall deformity due to a narrow A-P thoracic diameter. The latter may cause mechanical compression of cardiac chambers and a paradoxical septal motion, the so-called “septal bounce,” defined as the movement of the interventricular septum away from the LV free wall during systole, more enhanced during physical exercise [54]. If the exercise-induced septal bounce is not appropriately put in relation to the individual's anterior chest wall deformity, it may be misinterpreted as regional myocardial hypokinesis or dyskinesis due to obstructive CAD, based on expert echocardiographers also. It is likely that the mechanical stress induced by a narrow A-P chest diameter might have a preponderant or at least concurrent role in generating a dynamic LV dyssynchrony, which, in turn, is accentuated by physical exercise and secondary to compressive phenomena within the chest, rather than intrinsic myocardial dysfunction or true ischemia. A lower magnitude of LA A-P diameter/A-P thoracic diameter ratio (≤0.25) may help clinicians to quickly identify, among individuals with suspected CAD, those who have an increased probability to be diagnosed with FP-ESE.

To date, a great majority of studies have focused on the prognostic relevance of LA enlargement in CAD patients. It has been proposed that M-mode echocardiographic LA enlargement is a useful marker of abnormal ejection fraction, higher pulmonary capillary wedge and LV end-diastolic pressures, and triple-vessel disease in patients with CAD undergoing cardiac catheterization [11]. In addition, M-mode echocardiographic measurements of the left atrial dimensions were important markers of subclinical disease and conferred independent prognostic information for incident cardiovascular events, especially CAD, in elderly patients [12]. Moreover, in several studies performed during the last two decades, LAVi assessment was able to predict mortality and major adverse cardiovascular events in CAD patients, providing incremental prognostic information over M-mode-derived LA linear measurements [13-17]. The relation of LA enlargement with the risk of cardiovascular death may be partially mediated by increased LV mass. The increase in myocardial mass lowers coronary reserve, enhances cardiac oxygen requirements, and impairs LV filling and contractility [1,57].

Different from the above-mentioned studies that were all focused on the negative prognostic role of LA enlargement in the setting of CAD disease, our study revealed the potential protective role of a narrow LA A-P diameter corrected for the A-P thoracic diameter, i.e., the LA A-P diameter/A-P thoracic diameter ratio, as a surrogate indicator of FP-ESE result. Although current guidelines recommend to use LAVi as the main echocardiographic measurement of LA size [43], the LA A-P diameter corrected for the A-P thoracic diameter might still represent a major surrogate indicator in the clinical decision-making process, especially in the evaluation of patients with suspected CAD. Given that a lower magnitude of LA A-P diameter/A-P thoracic diameter ratio is a strong independent predictor of FP-ESE result, it is reasonable to shift the PTP of CAD from intermediate to low in all individuals with an LA A-P diameter/A-P thoracic diameter ratio ≤0.25, particularly in the younger age groups. Moreover, a flat/concave-shaped chest wall and a concomitant LA A-P diameter/A-P thoracic diameter ratio ≤0.25 might suggest clinicians to avoid ESE and/or further unnecessary exams (e.g., coronary computed tomography scan and/or coronary angiography), because of the excellent prognosis of these individuals. Finally, gender-specific M-mode reference values and nomograms of LA size, which are commonly reported as a function of height and BSA [58], should be probably adjusted for chest wall conformation also.

The main limitations of the present study are its retrospective nature, its monocentric design, and the limited sample size. Moreover, we analyzed only patients who had undergone ESE with positive results, and data from pharmacological stress echocardiography were not collected. However, this decision was in agreement with the current guidelines [43] that recommend ESE as the first choice for patients who are able to perform a physical exercise, because it is safer, more physiologic, and less expensive than other noninvasive imaging techniques [59]. Even if the analysis of LA strain can provide additional information beyond the LA volume in terms of prognostic risk stratification of CAD patients [17,60], the present study was not designed to assess LA reservoir strain by echocardiographic deformation imaging. Finally, given that only patients with positive ESE underwent coronary angiography, the sensitivity and specificity of ESE were not assessed.

## Conclusions

Echocardiographic assessment of the LA A-P diameter adjusted for the A-P thoracic diameter may allow clinicians to identify, among individuals with suspected CAD, those at lower risk of obstructive CAD. Accordingly, it could be appropriate to always perform an echocardiographic examination comprehensive of both A-P linear and volumetric measurements of LA size, corrected for anterior chest wall conformation.

Further retrospective or prospective studies with larger sample sizes could be designed to identify the best cut-off values of LA A-P diameter/A-P thoracic diameter ratio that are able to rule out obstructive CAD.
